# Preclinical Establishment of a Divalent Vaccine against SARS-CoV-2

**DOI:** 10.3390/vaccines10040516

**Published:** 2022-03-26

**Authors:** Zsofia Hevesi, Daniela Anna Gerges, Sebastian Kapps, Raimundo Freire, Sophie Schmidt, Daniela D. Pollak, Klaus Schmetterer, Tobias Frey, Rita Lang, Wolfgang Winnicki, Alice Schmidt, Tibor Harkany, Ludwig Wagner

**Affiliations:** 1 Center for Brain Research, Department of Molecular Neurosciences, Medical University of Vienna, 1090 Vienna, Austria; zsofia.hevesi@meduniwien.ac.at (Z.H.); tibor.harkany@ki.se (T.H.); 2 Division of Nephrology and Dialysis, Department of Medicine III, Medical University of Vienna, 1090 Vienna, Austria; daniela.gerges@meduniwien.ac.at (D.A.G.); sebastian.kapps@meduniwien.ac.at (S.K.); sophie.schmidt@akhwien.at (S.S.); wolfgang.winnicki@meduniwien.ac.at (W.W.); alice.schmidt@meduniwien.ac.at (A.S.); 3 Unidad de Investigacion, Hospital Universitario de Canarias-FIISC, 38320 La Laguna, Spain; rfreire@ull.edu.es; 4 Instituto de Tecnologías Biomedicas, Universidad de La Laguna, 38320 La Laguna, Spain; 5 Universidad Fernando Pessoa Canarias, 35450 Las Palmas de Gran Canaria, Spain; 6 Center for Physiology and Pharmacology, Department of Neurophysiology and Neuropharmacology, Medical University of Vienna, 1090 Vienna, Austria; daniela.pollak@meduniwien.ac.at; 7 Department of Laboratory Medicine, Medical University of Vienna, 1090 Vienna, Austria; klaus.schmetterer@meduniwien.ac.at (K.S.); tobias.frey@meduniwien.ac.at (T.F.); 8 Division of Endocrinology, Department of Medicine III, Medical University of Vienna, 1090 Vienna, Austria; rita.lang@meduniwien.ac.at; 9 Department of Neuroscience, Biomedicum 7D, Karolinska Institutet, 17177 Solna, Sweden

**Keywords:** receptor-binding domain, nucleocapsid, bivalent vaccine, neuroinflammation

## Abstract

First-generation vaccines against SARS-CoV-2 do not provide adequate immune protection. Therefore, we engineered a divalent gene construct combining the receptor-binding domain (RBD) of the spike protein and the immunodominant region of the viral nucleocapsid. This fusion protein was produced in either *E. coli* or a recombinant baculovirus system. Subsequently, the fusion protein was mixed with adjuvant and administered to mice in a prime-booster mode. Mice (72%) produced an IgG response against both proteins (titer: 10^−4^–10^−5^) 14 days after the first booster injection, which was increased to 100% by a second booster. Comparable IgG responses were detected against the delta, gamma and omicron variants of the RBD region. Durability testing revealed IgGs beyond 90 days. In addition, cytolytic effector cell molecules were increased in lymphocytes isolated from peripheral blood. Ex vivo stimulation of T cells by nucleocapsid and RBD peptides showed antigen-specific upregulation of CD44 among the CD4^+^ and CD8^+^ T cells of vaccinated mice. No side effect was documented in the central nervous system. Cumulatively, these data represent a proof-of-principle approach alternative to existing mRNA vaccination strategies.

## 1. Introduction

Many efforts have been made to generate spike glycoprotein-based vaccines to induce immunoprotection against SARS-CoV-2 infection. Both mRNA-based [1,2] and protein-based [3] vaccines are effective in humans. Further protein-based designs at the preclinical stage of development promise superior safety and efficacy, at least in animal models [4]. However, there has been and continues to be a growing concern that existing and emerging virus mutants may escape currently available vaccine-induced immunity [5]. Although vaccinated individuals infected with the delta or omicron variants of SARS-CoV-2 have a lower infectious viral titer (IVT) than non-vaccinated individuals [6], the spread of these variants is very rapid despite the high rates of vaccination using mRNA or vector vaccines encoding the spike protein alone. Therefore, several experimental and clinical vaccine studies have attempted to include other SARS-CoV-2 proteins that were expected to produce high levels of antibody titers and T-cell responses [7,8,9].

One of the four structural proteins ubiquitously required for viral replication is the nucleocapsid, which is immediately translated and expressed at excess quantities so that it is even secreted from infected cells into the blood. As a result, nucleocapsid detection is an effective means to screen infected individuals even before the onset of symptoms [7,10], particularly because antibody production against this protein is rapid and abundant [11,12]. The 100–300 aa region of the nucleocapsid induces the highest antibody titer in humans [13], which is attributed to its octapeptide structure homologous to the four hyperendemic seasonal coronaviruses [14]. Epitope profiling of SARS-CoV-2 antibodies with cross-reactivity to seasonal coronaviruses [15], together with experimental data showing that an antibody against the nucleocapsid can protect mice from lethal infection with a hepatitis virus [16], highlights the conceptual and technical potential of targeting this protein epitope. In addition, recent work in hamsters showed that immunization with attenuated intracellular replicating bacterium-vectored vaccines protects from the typical disease pathology caused by SARS-CoV-2 infection [17]. Alternatively, specific antibodies raised against the receptor-binding domain (RBD) of the spike protein can neutralize and inhibit the entry of SARS-CoV-2 into angiotensin-converting enzyme-2-expressing cells [18]. As such, the RBD is an immunodominant and specific target of SARS-CoV-2 antibody production in virus-infected individuals [19].

Here, we generated a fusion protein that combines the nucleocapsid (N100–300 aa) and the RBD of the spike protein (S300–685 aa) in one molecule. Using this construct, the durability of IgG production was examined over three months. The stimulation of T cells was assessed by quantitative PCR for cytolytic effector molecules in lymphocytes. Ex vivo stimulation of T cells by nucleocapsid and RBD peptides was measured by the upregulation of CD44 on CD4^+^ and CD8^+^ T cells in vaccinated and adjuvant-only treated mice. In addition, it was demonstrated that no side effects were observed, especially those occurring in the brain and those related to morbidity in people exposed to other SARS-CoV-2 vaccines [20,21,22].

## 2. Materials and Methods

### 2.1. Study Rationale

**Figure 1:**
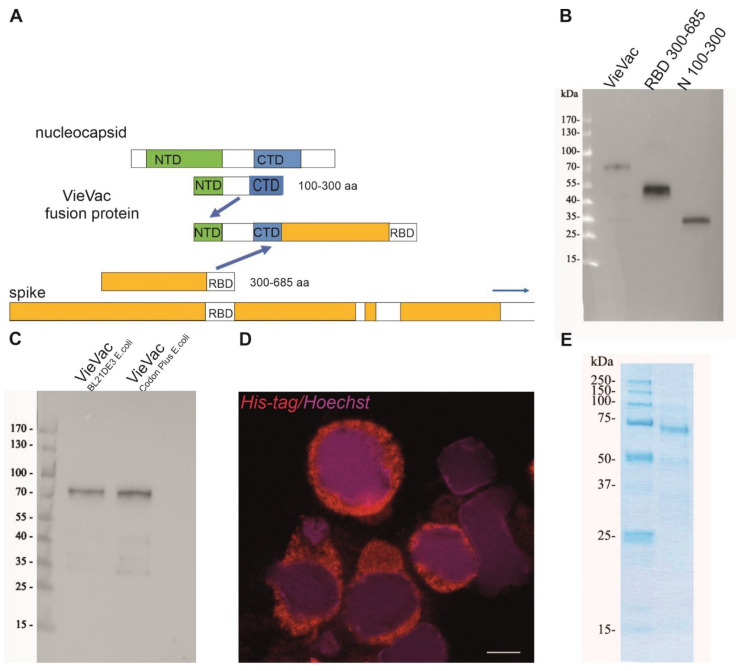
Expression construct and biochemical characterization of purified proteins. (**A**) Scheme of fusion protein design. The immunodominant region N100–300 aa of the nucleocapsid and S300–685 aa of the spike protein were fused, and the product (termed ‘VieVac’) was engineered into either the pET-30a *E. coli* expression vector or a baculovirus. (**B**) Protein immunoblot of *E. coli*-produced ‘VieVac’ fusion protein and its constituents using convalescent serum. The fusion protein (‘VieVac’, lane 1), S300–685 aa (lane 2) and N100–300 aa of the nucleocapsid protein (lane 3). Molecular weight marker is shown to the left. (**C**) Protein immunoblot of *E. coli*-produced fusion proteins ‘VieVac’ using mouse immune serum. The fusion proteins were purified out of *E. coli* lysate through their His-tag. VieVac produced in *E. coli* BL21DE3 (lane 1), and ‘VieVac’ produced in *E. coli* BL21Codon Plus (lane 2). (**D**) Immunofluorescence staining of Hi5 cells using anti-His.H8 mAb to detect ‘VieVac’ (red) in cells infected with baculovirus. Scale bar = 10 µm. (**E**) Protein staining of insect-cell-produced fusion protein ‘VieVac’. The fusion protein was purified through its His-tag out of Hi5 lysate results, with the full-size protein migrating at 72 kDa with only minor degradation products (lane 2). Molecular weight markers are shown to the left (lane 1).

Earlier work in infected individuals showed abundant antibody production against parts of the nucleocapsid, while neutralizing antibodies were shown to recognize regions in the RBD (and also NTD) of the spike protein [18,23]. These findings prompted us to design a fusion protein that included immunodominant regions of both the *S* and *N* proteins. Figure 1A and Figure S1 show the resultant divalent recombinant nucleotide and amino acid sequences, which we sought to test for their immunogenicity.

### 2.2. Animals, Blood Sampling and Tissue Processing

A total of 18 male and 4 female mice (C57BL/6J, 8–12-week-old) were group housed under standard conditions with a 12/12 light/dark cycle. The Austrian Federal Ministry of Education, Science and Research granted approval for the animal experiments (2022-0.169.722). All procedures conformed to the 2010/63 European Communities Council Directive. Mice were habituated for at least a week to their environments, and their numbers were kept at an absolute minimum. Blood was collected from the facial vein at a maximum volume of 200 µL every other week. At the end of the post-immunization survival period, mice were deeply anesthetized by isoflurane (at 5% with 1 L/min flow rate of tubed air) and then perfusion fixed by transcardially applying 4% (wt/vol) paraformaldehyde and 0.1% glutaraldehyde in 0.1 M phosphate buffer (PB; pH 7.0). Dissected brains were immersed in the same fixative (without glutaraldehyde) at 4 °C overnight. Brains were cryoprotected in 30% sucrose in PB at 4 °C for 3 days. Coronal sections (50 μm) were cut on a cryostat microtome (1-in-4 series) and kept in 0.05% NaN_3_ in PB until immunohistochemical processing.

### 2.3. Construction and Heterologous Expression of the Fusion Protein

The fusion protein was constructed using the Gibson assembly method [24]. For vector construction, the portion of the nucleocapsid (N100–300 aa) fused to the RBD (S300–685 aa), including 4 glycines as a hinge region, was cloned into a pET-30a vector and designated as ‘VieVac’ (Supplementary Figure S1A). This product was generated by first producing 2 fragments by PCR using the N and S cDNAs obtained from the Krogan laboratory as template [25]. The fragment containing the complete *N* protein portion and the beginning of the *S* protein were amplified with primers as follows: forward ATGGCTGATATCGGATCCGAATTCATGAAAGATCTCAGTCCGCGCTGG and reverse TTTAAGTGTACAACCACCGCCACCATGTTTGTAATCTGTCCCTTGCCG. To generate the second overlapping fragment containing the end of the *N* protein and the entire *S*-RBD sequence, we used the following primers: forward GATTACAAACATGGTGGCGGAGGTTGTACACTTAAAAGTTTTACGGTC and reverse GCCGCAAGCTTGTCGACGGAGCTCTCATCGCGCTCTTCGCGGGGAATT. In the final cloning step, the EcoR1 linearized pET-30a plasmid was combined with fragments 1 and 2.

### 2.4. Directional Cloning into the Gateway pEntry/D-TOPO Vector

A 4-nucleotide overhang (CACC) was placed in front of the forward primer CACCATGAAAGATCTCAGTCCGCG, while TCATCGCGCTCTTCGCGGGG served as reverse primer. The original construct (engineered pET-30a/VieVac) was used as template. The PCR conditions were as follows: 95 °C for denaturing, 58 °C for 30 s annealing and 72 °C for 2 min synthesis with 30 cycles. The resultant PCR amplicon was ligated into the pEntry/D-TOPO vector by incubation at 24 °C for 1 h. The resultant plasmid was transfected into One Shot TOP10 chemically competent *E. coli* using the heat shock method. Following 60 min incubation at 37 °C, *E. coli* were spread onto LB/kanamycin (50 µg/mL) plates and incubated at 37 °C overnight. Outgrowing colonies were amplified in 3 mL LB/kanamycin (50 µg/mL) in a shaking incubator (200/min) for 14 h. Plasmid isolation was carried out using the PureLink™ Quick Plasmid Miniprep Kit (Invitrogen, Waltham, MA, USA).

For detailed information on reagents, please refer to the list of materials and reagents in the Supplementary Materials. 

### 2.5. Insertion into Baculovirus and Amplification in Insect Cells

The pEntry/D-TOPO/VieVac construct (25 ng) was shifted into the baculovirus-compatible pDEST™ 10 expression vector. Using Clonase LR-Reaction II, both plasmids (pEntry/D-TOPO/VieVac and pDEST™ 10) were combined together with 1 µL 5× LR Clonase II enzyme in a final volume of 5 µL. This mixture was incubated at 25 °C for 3 h. Then, 1 µL was used to transform One Shot chemically competent *E. coli* by applying the heat shock method. Following 60 min incubation at 37 °C, the cells were spread onto LB/ampicillin (100 µg/mL) agar plates and incubated at 37 °C overnight. The outgrowing clones were amplified in liquid culture (LB/ampicillin, 100 µg/mL) with plasmids isolated using the PureLink Quick Plasmid Miniprep Kit. Following sequence verification, the Bac-to-Bac^®^ baculovirus system (Invitrogen) was chosen to generate the VieVac bacmid. In brief, Max Efficiency^®^ DH10Bac™ competent *E. coli* were transformed with the engineered pDEST™ 10 containing the VieVac recombinant construct using the heat shock method at 42 °C for 30 s. Transformants were grown at 37 °C under shaking in SOC medium for 4 h. A 10-fold serial dilution of the cells was made with each of them spread for selection on 7 µg/mL gentamicin-, 10 µg/mL tetracycline-, 50 µg/mL kanamycin-, 100 µg/mL BluoGal- and 40 µg/mL IPTG-containing LB agar plates. White colonies appearing after 48 h were re-plated and incubated at 37 °C for 14 h. Colonies with white phenotype were grown out in liquid culture to isolate recombinant bacmid DNA using the miniprep method. Following insert verification by PCR, Sf9 insect cells were transfected using Cellfectin^®^ II reagent. Then, 8 µL of Cellfectin^®^ was diluted in 100 µL Grace’s Insect Medium (unsupplemented) and 1 µg of recombinant baculovirus DNA in 100 µL Grace’s Insect Medium (unsupplemented). We then mixed diluted Cellfectin^®^ and baculovirus DNA, incubated them for 15 min at room temperature, and added them to the insect cells freshly plated 1 h earlier. The transfection medium was replaced by protein expression medium (SFM4Insect™ with L-glutamine). Protein expression was confirmed by a monoclonal antibody against the His-tag HIS.H8 (see Section 2.7).

### 2.6. ‘VieVac’ Purification from Insect Cells

Seventy-two hours after infection, Sf9 or Hi5 cells were centrifuged at 600× *g* for 5 min. Cells in the pellet were lysed using diluted BugBuster Protein Extraction Reagent^®^ (1:3 in PBS). After lysate sonication for 10 s and addition of benzonase endonuclease, the insoluble material was pelleted at 12,000× *g* at 4 °C for 10 min. The pellet was re-solubilized in 6 M GuHCl, 0.1 M NaH_2_PO_4_ and 0.01 M Tris-Cl (pH 8.0) and passed through a Ni-NTA binding column (Qiagen, Hilden, Germany). The loaded column was washed with 8 M urea, 0.1 M NaH_2_PO_4_ and 0.01 M Tris-Cl (pH 8.0), followed by 8 M urea, 0.1 M NaH_2_PO_4_ and 0.01 M Tris-Cl (pH 6.3) as also described for proteins produced in *E. coli* (see below). Finally, elution of the recombinant protein was carried out with 8 M urea, 0.1 M NaH_2_PO_4_ and 0.01 M Tris-Cl (pH 4.5) containing 250 mM imidazole. The resultant eluate was tested for protein content by spectrophotometry and immunoblotting.

### 2.7. Evaluation of Protein Expression by Immunofluorescence

In order to test ‘VieVac’ fusion protein expression in insect cells, Sf9 and Hi5 cells suspended in tissue culture medium were employed to generate cyto-slide preparations using a cyto-centrifuge. Air-dried cyto-preparations were fixed in acetone for 5 min. A water-repellent circle was drawn around the area where cells were spread. Following wetting with phosphate-buffered saline (PBS, pH 7.4), 60 µL of pre-diluted HIS.H8 mAb (Sigma, St. Louis, MO, USA) was applied onto the cells and incubated for 2 h under continuous agitation at room temperature in a moisturized chamber. After washing in PBS for 10 min, Alexa Fluor 594-tagged goat anti-mouse antibody was applied for 1 h at room temperature under continued gentle agitation. Hoechst 33,342 was routinely used as nuclear counterstain. Following repeated washes, specimens were mounted by Vectashield and coverslipped. Images were captured on a confocal microscope (Zeiss 880, Berlin, Germany).

### 2.8. Protein Production and Purification from E. coli

The engineered pET-30a/VieVac vector was transformed into BL21 (DE3) *E. coli* using the heat shock method: 2 µL of plasmid was mixed with 100 µL BL21 (DE3) or BL21 Codon Plus *E. coli* and incubated on ice for 30 min. Following a 45 s heat shock at 42 °C, the samples were returned to ice for 5 min. SOC medium was applied, and bacteria were incubated for 60 min at 37 °C under constant shaking. Aliquots were spread onto LB/kanamycin plates, which were incubated at 37 °C overnight. Kanamycin-resistant colonies were expanded in liquid culture using LB/kanamycin broth overnight. The culture volume was expanded 5× with Terrific Broth and induced with 200 µmol isopropyl-ß-D-thiogalactopyranoside (IPTG) to induce protein expression (at 22 °C over 24 h, shaking at 190/min). Protein extraction was performed in 8 M urea, 0.1 M NaH_2_PO_4_ and 0.01 M Tris Cl (pH 8), following pelleting of bacteria at 6000 rpm in a FIBERLite^®^ rotor (ThermoScientific, F15-8x50cy). The resultant bacterial lysate was centrifuged at 12,000× *g* for 10 min. Pre-cleared supernatant was mixed with Ni-NTA His-Bind Resin (Novagen, Madison, WI, USA) [13,14] and incubated under constant rotation (roller mixer SRT6D, Stuart^®^) for 10 min. The bacterial lysate/resin was loaded onto a Poly-Prep chromatography column (Bio-Rad, Hercules, CA, USA) and drained by unit gravity flow. The column was washed with 8 volumes of 8 M urea, 0.1 M NaH_2_PO_4_ and 0.01 M Tris Cl (pH 6.3) also containing 20 mM imidazole. Specifically bound recombinant protein was eluted in 8 fractions of 300 µL, each using elution buffer (8 M urea, 0.1 M NaH_2_PO_4_, 0.01 M Tris Cl (pH 4.5), 250 mM imidazole).

### 2.9. Immunoblotting of ‘VieVac’ Protein with Immune Sera

Aliquots of the purified protein or its *N* and *S* subregions [13,14] were loaded into individual wells of a 4–20% 10-well SDS-PAGE and run under reducing conditions. The gel was transferred by semidry blotting onto nitrocellulose membranes. The resultant filter was blocked in BM chemiluminescence blocking reagent for 30 min and incubated in 1:100 convalescent serum or 1:1000 mouse immune serum at room temperature for 2 h. Following 2 washes with TPBS (10 min each), goat anti-human IgG (H + L) F(ab’)2 HRP or goat anti-mouse immunoglobulin/HRP was applied and incubated at room temperature under constant shaking for 2 h. Following another 2 washes with TPBS (10 min each), antibody binding was detected by using BM chemiluminescence substrate solutions A and B. Images were recorded with the Fusion FX Vilber Lourmat (Vilber, Eberhardzell, Germany).

### 2.10. Protein Adsorption to Imject™ Alum Adjuvant

Next, we tested whether the recombinant protein could adsorb onto Imject™ Alum adjuvant [26]. The protein was mixed with the adjuvant in a 1:1 (*v*/*v*) ratio; then, the potentially formulated vaccine was dialyzed with a Slide-A-Lyzer dialysis cassette (Thermo Scientific, Waltham, MA, USA, MWCO 3500) against PBS at 4 °C under constant stirring for 4 h. To demonstrate the adsorption of the immunogen to the adjuvant, the nanostructured vaccine was centrifuged at 12,000× *g* for 4 min. The pre-cleared supernatant and pellet were each treated with Laemmli sample buffer, loaded separately onto a 12% SDS-PAGE gel and run under reducing conditions. The gel was transferred onto nitrocellulose membranes by semidry blotting. The filter was then incubated with convalescent sera diluted 1:100 and further developed as described above.

### 2.11. Mouse Immunization

**Figure 2:**
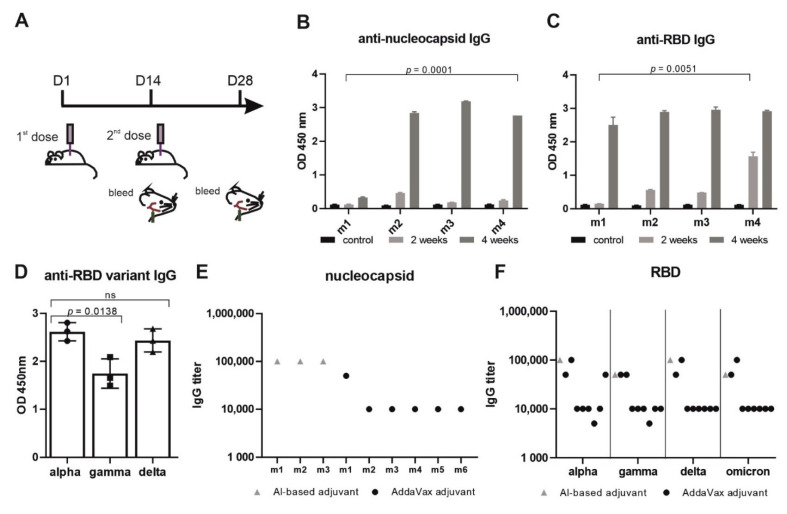
‘VieVac’ produces prolonged immune responses against all known virus strains. (**A**) Adult C57BL/6J mice (*n* = 4) were immunized with 20 µg Imject™ Alum/VieVac (i.p.), with a booster injection carried out 14 days later. Blood sampling was performed 1 h before immunization and 28 days after the first immunization. (**B**) Anti-nucleocapsid and (**C**) anti-spike IgG responses were measured by ELISA upon administration of Imject™ Alum/’VieVac’ in 4 mice (m1, m2, m3, m4) 2 weeks (gray) or 4 weeks after the first injection (dark gray) as compared to controls (black). (**D**) Anti-RBD IgG cross-reactivity in mice (*n* = 3) against the alpha variant, relative to the gamma and delta variants treated with the prime-booster mode of Imject™ Alum/’VieVac’ 90 days prior. (**E**) Anti-nucleocapsid IgG end-titer in Imject™ Alum/VieVac (gray) and AddaVax™/’VieVac’ (black) prime-booster-immunized mice. (**F**) Anti-RBD IgG end-titer (alpha, gamma, delta, omicron variants) in Imject™ Alum/’VieVac’ (gray) and AddaVax™/’VieVac’ (black) prime-booster-immunized mice.

The purified VieVac protein was mixed with Imject™ alum, and mice (*n* = 4) were injected intraperitoneally with 20 µg of the prepared immunogen after 5 h of dialysis against PBS. In the second step, the adjuvant AddaVax™ combined with the VieVac protein was similarly dialyzed; then, animals were injected with only the adjuvant for the control group (*n* = 5) and with the immunogenic AddaVax/VieVac protein at doses of 10 µg, 20 µg and 40 µg (*n* = 13). In both experiments, a second immunization with the same antigen preparation was administered after 14 days (Figure 2A). Serum was collected 14 and 28 days after the first immunization and tested by ELISA. Gene expression in blood cells and spleen was measured by qPCR at day 28.

### 2.12. ELISA

Ninety-six-well or three hundred eighty-four-well flat-bottom non-tissue-culture-treated plates (Falcon) were coated with 100 µL/well or 50 µL/well of recombinant protein, respectively (both N and S-RBD variants alpha, gamma, delta and omicron). A concentration of 1 µg/mL was chosen. After 1 h of incubation at room temperature on a plate shaker set to 300 rpm, the antigen-containing fluid was replaced by protein-containing blocking buffer (2× blocking reagent in PBS) for an incubation period of 30 min. Following a brief wash with PBS (200 µL), mouse serum diluted 10^−2^ to 10^−5^ in 100 µL assay buffer was incubated on a plate shaker for 60 min. The ELISA plate was then washed 3× with 350 µL TPBS using an ELISA washing machine (ELX50 Auto Strip Washer, Bio-Tek, Inc., Crawfordsville, IN, USA). Antibody binding was determined with goat anti-mouse-HRP conjugate (1:10,000 in assay buffer supplemented with goat serum (2% v/v)) and incubated at room temperature for 60 min. Finally, 100 µL of TMB substrate/chromogen mixture was applied and reacted in the dark for 10 min. Color development was terminated by adding 100 µL of 2 M H_2_SO_4_ and read on an ELISA reader (Synergy H1 Hybrid Reader, Bio-Tek, Inc.) at 450 nm. Each sample was processed in technical duplicates. *S* and *N* titers were measured by end-titration using flat-bottomed 384-well Nunc™ plates. ELISA was performed under similar conditions as above, with the reaction volume reduced to 50 µL per well. The reciprocal of the serum dilution was chosen, which gave a 2-fold OD value over the control.

### 2.13. RNA Isolation from Blood and Spleen

Peripheral blood was subjected to hemolysis using the erythrocyte lysis (EL) buffer (Qiagen), and RNA was isolated from white blood cells using the MagMAX mirVana Total RNA isolation kit. In brief, lysis buffer mixed with isopropanol was added to each cell pellet and incubated for 3 min. RNA-binding bead mix was added to adsorb RNA onto magnetic beads. Following aspiration of the supernatant, magnetic beads were washed sequentially. TURBO DNase solution was used to prevent genomic DNA contamination, followed by addition of rebinding buffer mixed with isopropanol. Magnetic beads were washed again and, after removing the supernatant, dried on a rotating platform. Finally, RNA was released by preheated elution buffer and evaluated for its concentration. Tissue fragments of 20–50 mg of mouse spleen were minced in Precellys Ceramic Kit 2.8 mm tubes using the Precellys 24 lysis and homogenization device (Bertin Technologies, Frankfurt am Main, Germany) following the addition of 1000 µL of TRIzol™ reagent. Subsequently, 200 µL of chloroform was applied and mixed by frequently inverting each tube. Following centrifugation at 12,000× *g* for 10 min at 4 °C, the aqueous phase was mixed with 500 µL isopropanol. After incubation at room temperature for 10 min, RNA was pelleted by centrifugation at 12,000× *g* for 15 min. RNA pellets were dried after a wash with 75% ethanol and dissolved in nuclease-free water for quantification using a Nanodrop device.

### 2.14. Quantitative PCR

Six hundred nanograms of RNA was mixed with random primers heated at 65 °C and briefly chilled on ice. dNTPs, RevertAid RT and reaction buffer were added and subjected to first-strand synthesis at 55 °C for 60 min, following primer annealing at 25 °C for 5 min. Enzyme activity was inactivated by heating at 80 °C for 10 min. A 20 µL first-strand solution was diluted with 60 µL nuclease-free water and either processed immediately or stored at −80 °C. For quantitative PCR, 2 µL of first-strand DNA, 1 µL of 10× gene-specific ProbeSet from TaqMan, 5 µL of TaqMan 2× universal PCR master mix and 2 µL H_2_O were mixed for each reaction, which were all carried out in duplicate. Reactions were performed on 384-well plates in a QuantStudio 6 Flex machine (Applied Biosystems, Waltham, MA, USA). In each experiment, 40 cycles were recorded, and quantitative data were presented in ΔΔCT mode and showing fold regulation relative to the control. Statistics were calculated using ΔCT values of individual samples.

### 2.15. Ex Vivo T-Cell Stimulation with N- and S-Specific Peptides and CD44 Evaluation by Cell Surface Flow Cytometry

Viable mouse splenocytes were isolated by homogenizing spleen tissues through a 70 µm mesh followed by erythrocyte lysis using the RBC lysis buffer (Biolegend, San Diego, CA, USA) according to the manufacturers’ instructions. Subsequently, splenocytes were seeded in flat-bottom 96-well plates in triplicate at 1 × 10^5^ cells per well. For antigen-specific stimulation, cells were pulsed with the PepTivator SARS-CoV-2 Prot_S Complete peptide mix (covering the complete sequence of the mature SARS-CoV-2 S protein) or the PepTivator SARS-CoV-2 Prot_N peptide mix (covering the complete sequence of the SARS-CoV-2 N protein; both Miltenyi Biotec). As controls, splenocytes were either left unstimulated or activated polyclonally with 12.5 µg/mL phytohemagglutinin (PHA). Cells were then cultured for five days in RPMI supplemented with 10% FCS and 10 µg/mL gentamycin. For analysis of activation, cells were harvested, and the respective triplicates were pooled. Cells were then stained with anti-mouse CD4 FITC (clone GK1.5), anti-mouse CD8 AlexFluor700 (cloneYTS156.7.7) and anti-mouse CD44 PerCP-Cy5.5 (clone IM7; all Biolegend) in PBS + 0.5% BSA + 0.05% NaN_3_. Expression of the activation marker CD44 was measured on a FACS Canto II flow cytometer (Becton Dickinson, Franklin Lakes, NJ, USA) and analyzed using FlowJo software v10 (TreeStar, Woodburn, OR, USA). For quantification, CD44^high^ cells in the CD4^+^ and CD8^+^ populations from the respective stimulation conditions were corrected against the unstimulated negative control.

### 2.16. Immunohistochemistry

Free-floating sections were rinsed in PB (0.1 M, pH 7.4). Non-specific immunoreactivity was suppressed by incubating the sections in a mixture of 5% normal donkey serum (NDS; Jackson ImmunoResearch, Baltimore Pike, PA, USA) and 0.3% Triton X-100 (Sigma) in PB at room temperature for 2 h. Sections were then exposed (4 °C for 3 days) to select combinations of primary antibodies (Supplementary Table S1) diluted in PB to which 0.1% NDS and 0.3% Triton X-100 were added. After extensive rinsing in PB, immunoreactivities were revealed by carbocyanine (Cy) 2-, 3- or 5-tagged secondary antibodies raised in donkey (1:500 (Jackson ImmunoResearch), at 22–24 °C for 2 h). Glass-mounted sections were coverslipped with Toluol-containing Entellan (Sigma). Sections were inspected, and images were acquired on a LSM880 confocal laser-scanning microscope (Zeiss) at either 10× or 63× primary magnification, with the pinhole set to 0.5–0.7 μm. Emission spectra for each dye were limited as follows: Cy2 (505–530 nm), Cy3 (560–610 nm) and Cy5 (650–720 nm). Cell counting was performed in ImageJ. Three sections of the hippocampus (CA1) were analyzed per animal (*n* = 3/experimental group). The stratum pyramidale was excluded from quantification because of its high neuron density.

### 2.17. Statistical Analysis

Adherence to a Gaussian distribution was determined using the Kolmogorov–Smirnov test. Normally distributed data are provided as means ± s.d. In cases of skewed distribution, data are described as medians (25th and 75th percentiles). Qualitative variables are described with counts and percentages and compared using Fisher’s exact test. A two-tailed *p*-value of 0.05 was considered statistically significant. Data were analyzed with SPSS^®^ Statistics (version 21). In histochemical experiments, data were normalized to a surface area of 1 mm^2^ and expressed as means ± s.e.m, followed by one-way ANOVA in GraphPad Prism.

## 3. Results

In this work, data are presented on a unique, novel, bioengineered fusion protein in *E. coli* and insect cells combining the RBD of the spike protein and the highly immunogenic part of the nucleocapsid protein. Efficacy, together with safety aspects, was tested in a mouse model.

### 3.1. Protein Production, Characterization and Purification

First, we produced and purified the fusion protein (termed ‘VieVac’) in *E. coli*. The recombinant protein containing a 6-His-tag was successfully purified by 8M urea. As shown in Figure 1B,C, the recombinant protein migrated at ~70 kDa, which is at the calculated cumulative molecular weight of the N100–300 aa (lane 3 in Figure 1B, 27.1 kDa according to calculations) and S300–685 (lane 2 in Figure 1B, 48,4 kDa according to calculations). In addition to the entire ‘VieVac’ protein, a truncated fragment co-purified in most experiments due to a premature translation stop (lane 2 in Figure 1C).

To evaluate whether the ‘VieVac’ protein would adsorb to an AlOH- and AlPO_4_-based adjuvant (Imject™ Alum), immunoblotting of formulated ‘VieVac’ was performed after the pelleting of the nanoparticulate structures by centrifugation. This revealed that the protein was recovered in its entirety in the particle-containing pellet (Figure S2, lane 3).

In a second attempt, ‘VieVac’ production was performed in eukaryotic cells. This was motivated by the fact that the RBD is structurally composed of a twisted five-stranded antiparallel β-fold, with strands and loops connecting the β-strands, and is kept in its configuration by four disulfide bonds between eight cysteines. Since this delicate type of folding develops better in eukaryotic cells, the recombinant gene fusion was re-engineered into the baculovirus system with the non-truncated protein successfully produced in Hi5 cells as demonstrated by immunofluorescence labeling 72 h after infection (Figure 1D). This protein could be purified through its N-terminal His-tag in its full length. Only minor degradation products co-purified on Ni-NTA columns (Figure 1E).

### 3.2. Imject™ Alum/’VieVac’ Induces IgGs to Both Nucleocapsid and RBD

To reduce the required amount of the antigen, the recombinant fusion protein was adsorbed onto the AlOH- and AlPO_4_-based adjuvant. First, mice (*n* = 4) were challenged with this immunogen in a standard regimen (Figure 2A) [27]. Immunogen-challenged mice developed IgG within 14 days (nucleocapsid titer 10^−2^–5 × 10^−2^ and RBD 10^−2^–10^−3^) after the first dose, which increased to a significant IgG response to both antigens 28 days after the initial injection (nucleocapsid end-titer 10^−5^ and RBD end-titer 5 × 10^−4^–10^−5^) (Figure 2B–F). None of the mice showed any adverse side effects, e.g., weight loss or neurological complications (data not shown). Mouse #1 had a shallow IgG response toward the nucleocapsid after the first booster injection (end-titer 10^−2^) (Figure 2B). When receiving a second booster, this mouse also responded to the nucleocapsid when tested later (Figure 2D) with an end-titer of 10^−5^ (Figure 2E).

In order to investigate the durability of the IgG immunogen response, mice were kept under normal conditions and tested after 90 days for the mutant RBD of SARS-CoV-2 variants termed gamma (K417T and E484K) and delta (L452R and T478K). This revealed immune cross-reactivity of alpha RBD with the delta variant and significantly (*p* = 0.0138, 19%) reduced cross-reactivity with the gamma variant (Figure 2D). Mouse #1, having received two booster injections, showed similar reactivity toward both the alpha and delta variants.

### 3.3. AddaVax™ with ‘VieVac’ Primes IgG Production to Both Nucleocapsid and RBD

**Figure 3:**
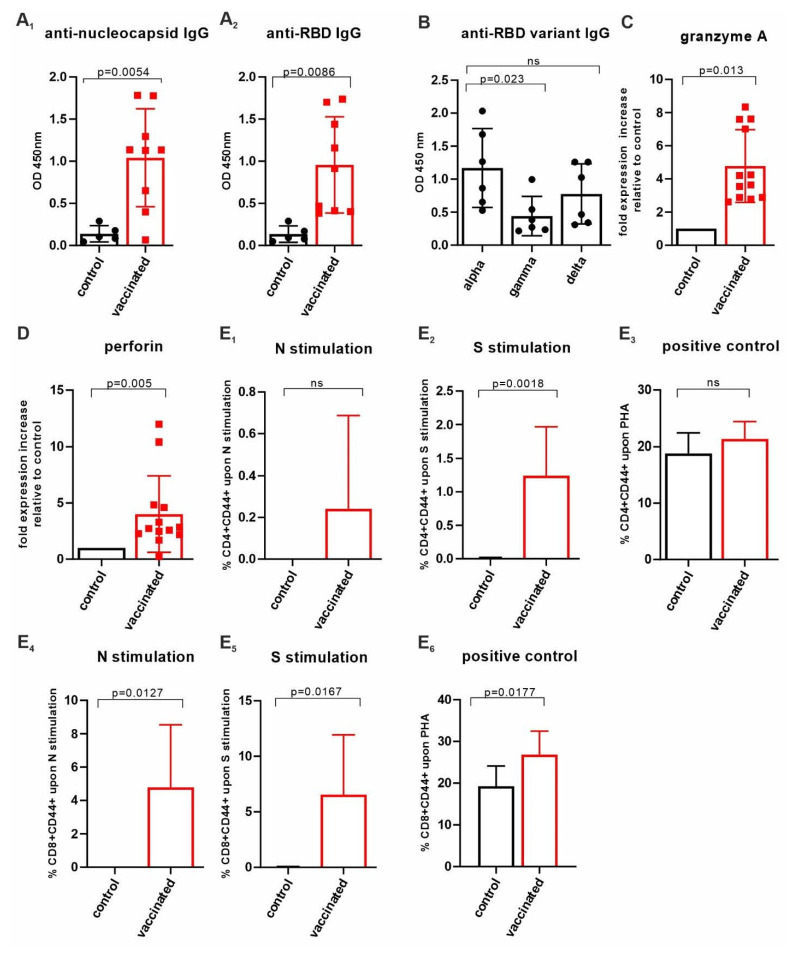
Assessment of cellular immunity upon AddaVax™/’VieVac’ administration. (**A_1_**,**A_2_**) AddaVax™/’VieVac’ immunization in prime-booster mode and IgG responses in 9 out of 13 mice. Control mice (*n* = 5) received AddaVax™ only, and test animals received AddaVax™/’VieVac’ containing 10–40 µg protein. (**B**) Cross-reactivity against SARS-CoV-2 RBD variants, alpha vs. gamma and delta. Sera of *n* = 6 prime-booster-injected mice with AddaVax™/’VieVac’ were tested for IgGs recognizing alpha as compared to gamma and delta versions of the RBD. (**C**,**D**) Effector molecule gene expression in peripheral blood. Granzyme A (**C**) and perforin (**D**) expression in peripheral lymphocytes was evaluated by qPCR using a TaqMan probe set. Relative fold expression is indicated in 12 AddaVax™/’VieVac’ prime-booster-injected mice in relation to the mean of *n* = 5 control mice that had received AddaVax™ adjuvant only. (**E_1_**–**E_6_**) CD4^+^ and CD8^+^ T-cell responses in ex vivo-stimulated spleen cells. CD4^+^ T cells (**E_1_**–**E_3_**) were stimulated ex vivo with N-specific (**E_1_**) and S-specific peptides (**E_2_**) or PHG (**E_3_**). Control mice (*n* = 5, adjuvant only) were compared to vaccinated mice (*n* = 13, AddaVax™/’VieVac’). CD8^+^ T cells (**E_4_**–**E_6_**) were stimulated ex vivo with N-specific (**E_4_**) and S-specific peptides (**E_5_**) of PHA (**E_6_**). Control mice (*n* = 5, adjuvant only) were compared to vaccinated mice (*n* = 13, AddaVax™/’VieVac’).

In a second study, ‘VieVac’ generated in a eukaryotic expression system was formulated with AddaVax™, an MF59-compatible adjuvant, which is approved in Europe and has been used in influenza vaccines [28] and beyond [29]. Mice (*n* = 13) were again challenged with three different doses of the recombinant divalent antigen mixed with AddaVax™ according to the benchmarked immunization strategy used above (see Figure 2A). Ten out of thirteen recipients produced IgG against both proteins (nucleocapsid titer: 10^−2^–10^−3^ and RBD titer: 10^−2^–10^−3^) after 14 days. Control mice received AddaVax™ only (*n* = 5). Fourteen days after a booster injection, 9 out of the 13 test mice showed a further increase in their IgG responses to both antigens (nucleocapsid titer: 10^−4^–5 × 10^−5^ and RBD titer: 5 × 10^−3^–10^−5^) (Figure 2E,F and Figure 3A_1_,A_2_).

Next, we tested mouse sera (*n* = 6) for individual IgG cross-reactivity against the virus variants gamma and delta. As compared to alpha, each serum IgG content was tested 14 days after the booster injection for its reactivity against the mutant RBD (Figure 3B). The immune response (per IgG) varied amongst the mice but was reduced on average by 63% (*p* < 0.023) toward the gamma variant and 43% (non-significant) toward the delta variant as compared to the alpha variant when measured at a titer of 10^−2^. However, similar end-titers were found for alpha, gamma/delta and omicron variants, ranging from 5 × 10^−3^ to 10^−4^, with the exception of one mouse in which the end-titer was an order of magnitude lower when alpha was compared with gamma, delta and omicron (Figure 2F). These data show that our VieVac’s strategy is equally efficient against all prevalent SARS-CoV-2 strains.

### 3.4. Insect-Cell-Produced AddaVax™/VieVac Generates Effector Cell Immunoreactivity

Next, we addressed the effector potential of the constructs injected by analyzing cytotoxic lymphocytes (NK-T cells), which have been suggested to be the most important determinants of cellular anti-SARS-CoV-2 immunity. In peripheral blood, the AddaVax™/VieVac immunized group (*n* = 13) showed a 4.6-fold (*p* < 0.013) granzyme A and a 4-fold (*p* < 0.005) perforin mRNA increase, as compared to the AddaVax-only control group (*n* = 4). (Figure 3C,D). To investigate changes in the lymphocyte populations in the spleen, 20-25 mg of spleen tissue was homogenized, and RNA was extracted. When analyzed for changes in target gene expression, changes did not reach the level of significance, even though an upregulation in granzyme A expression was seen (Supplementary Table S2).

### 3.5. CD4^+^ and CD8^+^ T-Cell Responses Evaluated by CD44 Cell Surface Upregulation in Ex Vivo-Stimulated Spleen Cells of AddaVax™-/VieVac-Challenged Mice

N-specific and S-specific T-cell responses were measured following 5 days of ex vivo spleenocyte stimulation using T-cell-specific peptides of SARS-CoV-2 *N* and *S* proteins. Spleen T cells were obtained 14 days after the booster injection from mice injected with AddaVax™/VieVac (*n* = 13) as the test group and adjuvant (AddaVax™) only (*n* = 5) as the control group. The number of CD4^+^ T cells expressing CD44 upon stimulation by N-specific peptides was elevated in 7 mice out of 13 (Figure 3E_1_) but did not reach statistical significance when compared to that of the control group. Stimulation with *S*-specific peptides resulted in a significant increase in CD44 in the test group compared with the control group (*p* = 0.0018; Figure 3E_2_). The extent of activated CD8^+^ T cells after stimulation with N-specific peptides (*n* = 13) and *S*-specific peptides (*n* = 13) was significant relative to the control group (AddaVax™ only, *n* = 5; *p* = 0.0127 and *p* = 0.0167, respectively) (Figure 3E_4_,E_5_). Phytohemagglutinin (PHA) stimulation was used as a positive control to illustrate the spectrum of the CD44 response to a non-specific T-cell stimulant (Figure 3E_3_,E_6_). *S*-specific-stimulated CD8^+^ T cells showed significantly higher stimulation by PHA in terms of CD44 upregulation compared to the control group (*p* = 0.0177) (Figure 3E_6_). This suggests that a higher state of alertness to non-specific stimuli is induced within CD8^+^ T cells after S-specific peptide exposure.

### 3.6. Lack of Adverse Neuropathology in Addavax™-/VieVac-Injected Mice

Neurological side effects have been observed following vaccination with authorized viral vector-based vaccines against SARS-CoV-2 [20,22,30,31]. In particular, cerebrovascular venous and sinus thrombosis (CVST) is a subtype of stroke in which blood clots form and obstruct blood flow in the brain’s vascular system [32]. These conditions combined with a COVID-19-vaccine-related thrombocytopenia have been termed vaccine-induced immune thrombotic thrombocytopenia (VITT) [22,33], and this has become one of the most important research areas of our time.

**Figure 4:**
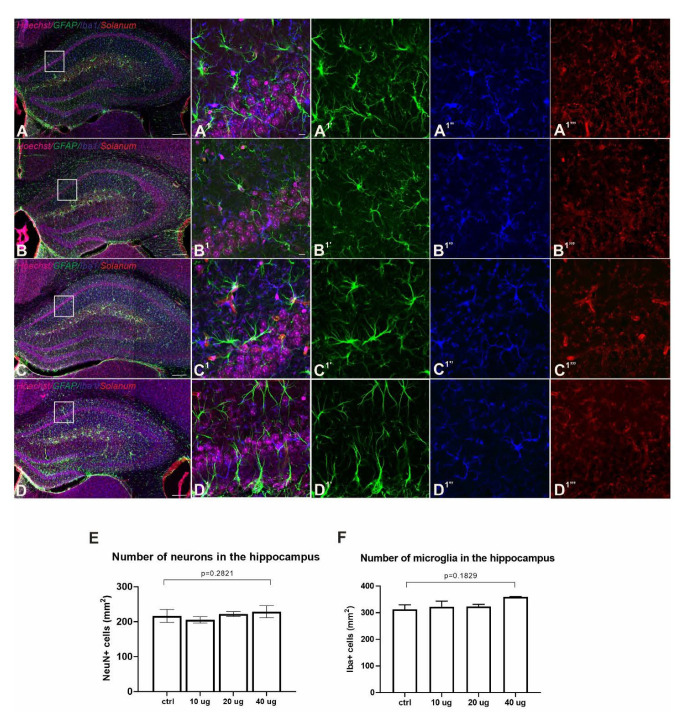
Lack of adverse side effects in the nervous system of immunized mice. (**A**–**D** and **A^1^**–**D^1^**) Fluorescence immunohistochemistry showed no accumulation in GFAP (green, astrocytes, (**A^1^′**–**D^1^′**)), Iba1 (blue, microglia, (**A^1^″**–**D^1^″**)) and *Solanum tuberosum* lectin (red, vasculature, (**A^1^‴**–**D^1^‴**)) distribution between control (**A**) and Addavax™-/’VieVac’-injected animals ((**B**) 10 µg, (**C**) 20 µg, (**D**) 40 µg). (**E**,**F**) Quantitative analysis of neuronal numbers (NeuN; (**E**)) and Iba1-positive microglia (**F**) in the hippocampus (CA1 subfield) showed no difference between the control and vaccinated groups. Scale bars = 200 µm (**A**–**D**), 10 µm (**A^1^′**–**D^1^‴**).

No differences were detected in endothelial morphology or blood vessel density/distribution between experimental groups (Figure 4A–D) [34]. Reduced capillary staining, the presence of plaques and branched (activated) microglia were also not detected. Likewise, the distribution, density and morphology of microglia and astrocytes also remained unchanged (Figure 4A–D) [35,36]. No differences were observed in the cell number of neurons in selected brain areas either (Figure 4E,F and Figure S4). These data support the notion that ‘VieVac’, at least in preclinical models, is unlikely to cause adverse neuropathological side effects.

## 4. Discussion

This study provides a proof-of-concept approach for the generation of divalent (or even polyvalent) protein backbones for the development of protein vaccines against SARS-CoV-2, taking advantage of the coincident presence of the most immunogenic viral regions hinged by flexible linkers to maintain ternary and quaternary structures. The construct design incorporates a rapidly mutating region (RBD) and a constitutive region (nucleocapsid), thus overcoming strain-specific hindrances in immunogenicity as increasingly seen for linearized mRNA vaccines. This suggests potent antibody production also against the omicron version of the RBD. Protein-based vaccines have not yet been tested for evoking cross-reactive antibody generation in humans.

The RBD region is the backbone of all vaccination strategies currently available, even if its inferiority upon the rapidly mutating SARS-CoV-2 is already apparent. Since conformation-specific antibody responses may be important in immune defense against SARS-CoV-2, recombinant proteins generated in eukaryotic systems are the tools of choice. This is significant because the RBD domain has a twisted five-stranded antiparallel ß-fold, with strands and loops connecting its ß-strands. This fold could be resolved by crystallography of the RBD produced in Hi5 insect cells [37]. There are nine cysteines in this region, eight of which are involved in disulfide bond formation in order to generate the ACE-2 binding structure. Here, we show that insect bioreactor systems could also be amenable to producing vaccine backbones. In particular, our molecular design incorporates eight cysteines from the RBD region (300–685 aa), thus likely stabilizing the ternary structure of the protein to increase its recognition by the host’s immune system, a feature likely contributing to the near-maximal immune response already at the second booster stage. In contrast, relatively limited emphasis has been placed on the well-transcribed nucleocapsid protein in the presently pursued SARS-CoV-2 vaccine strategies in Europe and beyond, even if the nucleocapsid of other coronaviruses was earlier recognized for its immunogenicity [38,39]. This lack of interest is surprising since 11% of human CD4^+^ T cells and 12% of CD8^+^ T cells recognize the nucleocapsid in individuals with SARS-CoV-2 infection [40], supporting its immunogenic potential to generate potent immunoprotection by expanding surveillance to cellular branches of defense in humans.

A homologue of an EDA-approved adjuvant was used to increase efficacy, which was, in terms of T-cell response, nearly complete after the second booster, similarly to others [29], and it reached 100% upon the third inoculation in a staggered primer-booster regimen. The fact that high immunogenicity is detected even after 90 days in a mouse immunization model with near-equivalent efficacy against the alpha, delta and omicron strains when measuring the end-titer gives confidence in the correctness of this design strategy. Immunological analysis of lymphocytes harvested from the spleen of immunized animals showed a significant T-cell response as indicated by the significant upregulation of granzyme A and perforin, cytotoxic granule effector molecules, in peripheral lymphocytes. An increased expression of CD44 upon N- and S-specific peptide stimulation ex vivo demonstrated the generation of T cells capable of executing cellular protection and the production of interleukins [41].

Given the brevity of time for our compressed proof-of-concept workflow, we recognize that data on the generation of neutralizing antibodies, whose presence is assigned to immunity against the RBD (‘spike’) protein, are, to date, lacking. However, and equally importantly, cellular immunity through T-cell responses is recognized as an essential means of protection. Accordingly, the adoptive transfer of T cells into immunodeficient mice led to rapid recovery after the transfer of SARS-CoV-specific effector cells [42]. Similar results are seen in ferrets, in which neutralizing antibodies do not fully protect against SARS-CoV-2-induced disease. Instead, nasal immunity, which is reliant on T-cell responses, together with the presence of antibodies, seems to carry optimal protection [43]. Notably, nucleocapsid-related B-cell immunity has also been shown in earlier studies [12,13,14,39,44], and it even served as a diagnostic tool because the detection of nucleocapsid-specific IgG in conjunction with RBD-specific IgG differentiated SARS-CoV-2 exposure from a pure mRNA-based vaccine response. Therefore, the pronounced B-cell responses shown here support the hypothesis that both viral protein fragments of our divalent construct can provoke significant cellular immunity. Considering that immunogenic peptides are native viral sequences (and neither forward nor reverse-engineered fragments that could curb immunogenicity), we view the B- and T-cell responses described here as minimally required yet sufficient experimental indices of our molecular design strategy to trigger significant protection against SARS-CoV-2.

## 5. Conclusions

We propose that the above molecular design, together with biological data on the efficacy of the administered divalent recombinant protein, outlines a rational approach to generate an efficient protein vaccine pipeline (which we term as a prototypic ‘VieVac’ vaccine), which can be built on both eukaryotic and prokaryotic bioreactors for scalable production. Bivalent protein-/adjuvant-based immunization is effective in eliciting humoral and cellular immunoresponses upon application in a prime-booster mode. Given that the repeated administration of ‘VieVac’ evoked no side effects in the nervous system, we emphasize the safety of a protein-based vaccine.

## Data Availability

All data and protocols relevant to this study are provided herein. Additional requests for materials (e.g., plasmids) can be made to the corresponding authors.

## Supplementary Materials

The following are available online at https://www.mdpi.com/article/10.3390/vaccines10040516/s1, Figure S1: ‘VieVa’ coding (A) and translated sequence (B), Figure S2: Adsorption of recombinant ‘VieVac’ protein to Imject™ Alum and immunoblotting with convalescent serum, Figure S3: Multiple immunofluorescence labelling confirmed the lack of adverse effects on the density and morphology of neurons and microglia in the hippocampus of immunized mouse brain, Figure S4: Multiple immunofluorescence labelling confirms the lack of inflammation in immunized mouse brain, Table S1: Antibodies and their use for biochemistry and histochemistry, Table S2: Gene expression in spleen measured by qPCR, List of material and reagents used.
