# Supplementary material for: Preclinical Establishment of a Divalent Vaccine against SARS-CoV-2
Source: Vaccines (Basel). 2022 Mar 26;10(4):516. doi: 10.3390/vaccines10040516 (PMC9028954; doi:10.3390/vaccines10040516)
Supplement: Supplementary file 1 [file vaccines-10-00516-s001.zip › vaccines-1649194-supplementary.pdf]

Supplementary information to the manuscript:

Preclinical establishment of a divalent vaccine against SARS-CoV-2

Zsofia Hevesi, Daniela Gerges, Sebastian Kapps, Raimundo Freire, Sophie Schmidt, Daniela D. Pollak, Klaus Schmetterer, Tobias Frey, Rita Lang, Wolfgang Winnicki, Alice Schmidt, Tibor Harkany, Ludwig Wagner

This file contains:

- Supplementary Figures S1-S4
- Supplementary Tables S1-S2
- List of material and reagents used

Supplementary Figure S1

'VieVa' coding (A) and translated sequence (B)

A. 100-300 (*N*) + 4x Gly + 300-685 (*S*)

ATGAAAGATCTCAGTCCGCGCTGGTACTTTTATTATTTGGGAACCGGCCCAGAAGCGGGCCTCCC
ATATGGAGCCAATAAGGACGGGATTATATGGGTGGCTACAGAGGGAGCCCTCAATACGCCAAAA
GATCACATCGGAACAAGAAACCCTGCTAATAATGCCGCAATCGTGCTGCAGTTGCCTCAGGGGAC
GACGCTTCCTAAAGGCTTTTACGCAGAAGGATCACGCGGCGGCAGCCAAGCATCCTCAAGGTCTA
GTTCCAGAAGTCGAAACAGCTCCAGAAACTCCACACCAGGGTCCAGTAGGGGCACAAGTCCGGC
GCGGATGGCGGGCAACGGCGGAGACGCCGCACTCGCTCTGTTGCTCCTGGACCGGCTCAACCAAC
TTGAATCCAAGATGAGTGGTAAGGGACAGCAGCAACAAGGTCAAACCGTAACCAAGAAAAGCGC
TGCAGAAGCTTCTAAAAAACCTCGACAAAAACGGACCGCTACGAAGGCATATAACGTTACTCAA
GCCTTCGGGAGACGAGGGCCGGAGCAAACCCAGGGGAATTTTGGAGATCAGGAGCTCATCCGGC
AAGGGACAGATTACAAACATGGTGGCGGAGGTTGTACACTTAAAAGTTTTACGGTCGAAAAGGG
AATCTACCAAACATCAAATTTTAGGGTACAACCTACGGAATCTATCGTACGCTTCCCGAATATCAC
TAACTTGTGTCCGTTCGGCGAGGTTTTTAATGCGACCAGGTTTGCTTCCGTGTACGCCTGGAACAG
GAAACGGATCTCCAATTGTGTCGCCGATTACTCCGTCTTGTATAATTCAGCATCTTTCAGCACGTT
TAAATGTTACGGAGTTTCCCCCACAAAATTGAATGACCTTTGCTTTACGAACGTCTACGCGGATTC
ATTTGTAATCCGGGGGGACGAAGTTAGGCAAATTGCGCCAGGGCAGACTGGCAAGATAGCTGAC
TATAATTATAAATTGCCGGATGACTTTACGGGCTGTGTGATTGCTTGGAACTCAAATAATCTGGAC
TCAAAGGTAGGGGGAAATTATAACTACCTTTACAGGCTGTTCCGGAAGAGTAATCTGAAGCCATT
CGAAAGAGATATAAGTACAGAGATCTACCAAGCTGGAAGCACCCCCTGCAATGGTGTTGAAGGA
TTCAATTGTTATTTCCCATTGCAATCCTATGGTTTTCAACCGACGAATGGGGTGGGATACCAACCA
TATCGAGTTGTGGTTCTCAGTTTCGAGTTGCTTCATGCTCCTGCGACAGTATGTGGACCAAAAAAA
TCTACTAATCTGGTGAAGAATAAATGCGTCAATTTTAATTTTAATGGTCTGACAGGTACCGGAGTG
CTTACGGAATCTAACAAGAAGTTTCTTCCTTTTCAACAGTTCGGGAGGGACATAGCCGACACAAC
TGACGCGGTAAGAGATCCGCAGACGTTGGAAATTCTCGATATAACTCCATGCTCATTTGGAGGAG
TGAGTGTTATCACTCCTGGTACGAACACCAGTAATCAGGTTGCAGTTCTTTATCAGGACGTGAATT
GTACAGAGGTCCCGGTTGCGATACACGCGGACCAGCTGACGCCCACGTGGCGGGTTTACTCAACT
GGGTCAAATGTGTTTCAAACCAGAGCCGGCTGCCTCATAGGAGCCGAGCATGTGAACAACAGTTA
TGAATGTGACATACCTATCGGGGCGGGGATCTGCGCTTCATATCAAACGCAAACCAATTCCCCGC
GAAGAGCGCGATGA

B.

(his Tag)-

MKDLSPRWYFYYLGTGPEAGLPYGANKDGIIWVATEGALNTPKDHIGTRNPANNAAIVLQLPQGTTL
PKGFYAEGSRGGSQASSRSSSRSRNSSRNSTPGSSRGTSPARMAGNGGDAALALLLLDRLNQLESKMS
GKGQQQQGQTVTKKSAAEASKKPRQKRTATKAYNVTQAFGRRGPEQTQGNFGDQELIRQGTDYKHG
GGGCTLKSFTVEKGIYQTSNFRVQPTESIVRFPNITNLCPFGEVFNATRFASVYAWNRKRISNCVADYS
VLYNSASFSTFKCYGVSPTKLNDLCFTNVYADSFVIRGDEVRQIAPGQTGKIADYNYKLPDDFTGCVIA
WNSNNLDSKVGGNYNYLYRLFRKSNLKPFERDISTEIYQAGSTPCNGVEGFNCYFPLQSYGFQPTNGV
GYQPYRVVVLSFELLHAPATVCGPKKSTNLVKNKCVNFNFNGLTGTGVLTESNKKFLPFQQFGRDIAD
TTDAVRDPQTLEILDITPCSFGGVSVITPGTNTSNQVAVLYQDVNCTEVPVAIHADQLTPTWRVYSTGS
NVFQTRAGCLIGAEHVNNSYECDIPIGAGICASYQTQTNSPRRAR*

Supplementary Figure S2

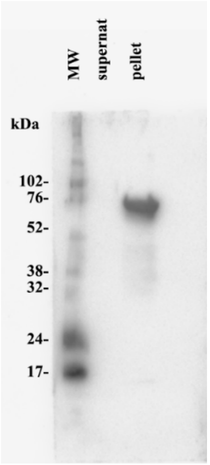

Adsorption of recombinant 'VieVac' protein to Imject™ Alum and immunoblotting with convalescent serum. Equal volumes of fusion protein solution and Imject™ Alum were incubated at room temperature for 5 min under constant rotation. An aliquot (20 µl) was centrifuged at 12,000 *g*. Pre-cleared supernatant (lane 2) did not contain any 'VieVac' protein but all was adsorbed onto the pelleted particles of Imject™ Alum (lane 3). Molecular weight (MW) markers are in lane 1.

Supplementary Figure S3

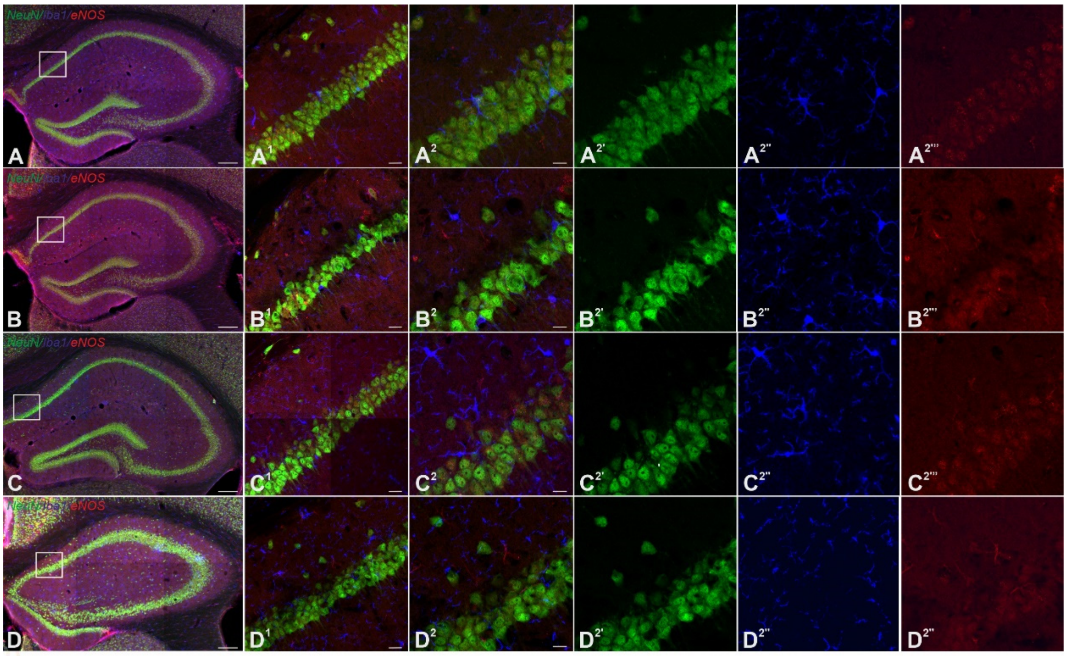

Multiple immunofluorescence labelling confirmed the lack of adverse effects on the density and morphology of neurons and microglia in the hippocampus of immunized mouse brain. NeuN (green, neurons, A2’-D2’), Iba1 (blue, microglia, A2’’-D2’’) and eNOS immunoreactivities (red, vasculature, A2’’’-D2’’’) were assessed in control (A) and Addavax™/’VieVac’-injected animals (B: 10 µg, C: 20 µg, D: 40 µg). *Scale bars* = 200 µm (A-D), 20 µm (A^1-D^1), 10 µm (A^2-$D^{2''}$).

Supplementary Figure S4

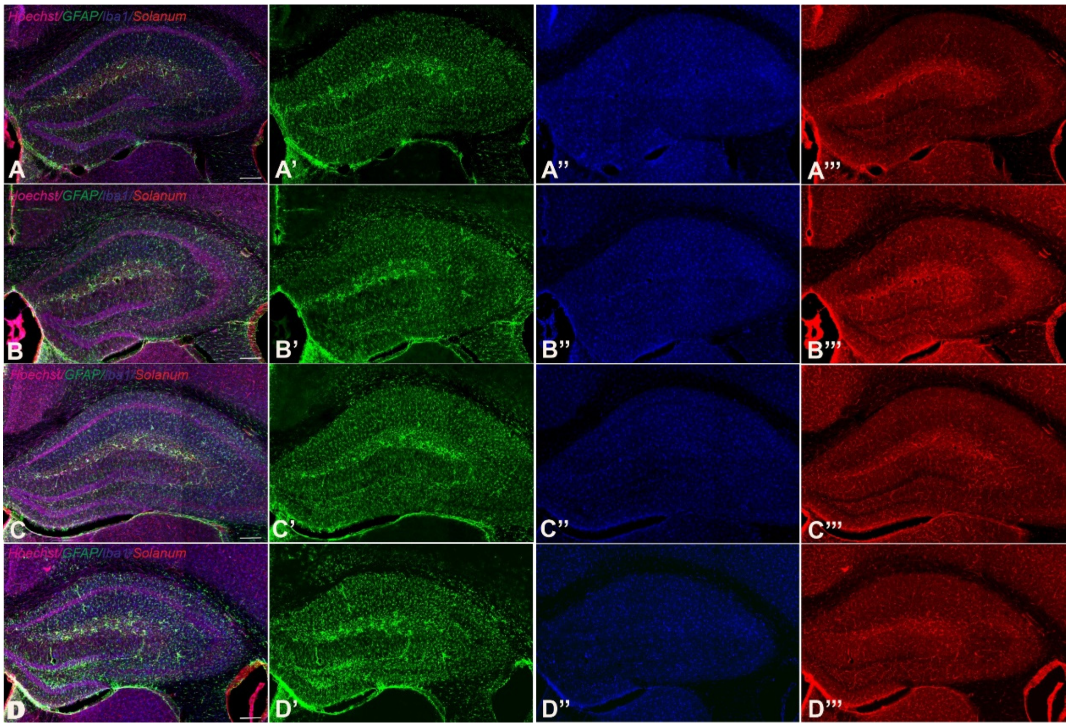

Multiple immunofluorescence labelling confirms the lack of inflammation in immunized mouse brain. GFAP (green, astroglia, A2'-D2'), Iba1 (blue, microglia, A2''-D2'') and eNOS immunoreactivities (red, vasculature, A2'''-D2''') were assessed in control (A) and Addavax™/'VieVac'-injected animals (B: 10 µg, C: 20 µg, D: 40 µg). *Scale bars* = 200 µm.

Supplementary Table S1

Marker	Company	Host	IH dilution	Catalogue no.
eNOS	Invitrogen	rabbit	1:300	PA3-031A
GFAP	Synaptic System	guinea pig	1:1000	173004
Iba1	Abcam	goat	1:500	Ab5076
NeuN	EMD Millipore	chicken	1:1000	ABN91
Solanum tuberosum biotinylated lectin	Vector Laboratories	-	10 µg/µl	B-1165-2
Hoechst 33,342	Sigma	-	1:10.000	23491-52-3
His tag HIS.H8	Millipore	mouse	1:500	05-949
anti-human IgG (H+L) F(ab')2 HRP	Millipore,	goat	1:20.000	AQ112P
anti-mouse immunoglobulin/HRP	Dako	goat	1:10.000	P0447

Antibodies and their use for biochemistry and histochemistry.

Supplementary Table S2

**Target Name**	**probe set, TaqMan**	**P value**
VEGFA	Mm01281447_m1	0,5953
CD19	Mm00515420_m1	0,7370
CD3e	Mm1179194_m1	0,7627
CD8a	Mm01182108_m1	0,6222
Granzyme A	Mm01304452_m1	**0,3351**
TGFb1	Mm03024053_m1	0,8565
IFNg	Mm01168134_m1	0,8044
Perforin	Mm00812512_m1	0,5776

Gene expression in spleen measured by qPCR in control (AddaVax adjuvant only, *n* = 4) *vs.* immunized mice (Addavax™/'VieVac', *n* = 13) 28 days after the initial injection. No statistical difference in gene expression was found. A trend towards up-regulation in granzyme A was seen in the test group.

List of material and reagents used.

	Company	Identifier
**Experimental models**		
BL21 (DE3) Competent Cells	Agilent Technologies	#230280
One Shot™ TOP10 Chemically Competent E. coli	Invitrogen	#C404010
High Five Insect cells (Hi5)	Invitrogen	#B855-02
Sf9 cells in Sf-900™ II SFM	Gibco	#11496015
pEntry/D-TOPO vector	Invitrogen	# CA92008
One Shot TOP10 chemically competent E.coli	Invitrogen	# CA92008
PureLink™ Quick Plasmid Miniprep Kit	Invitrogen	# K210010
pDEST™ 10	Invitrogen	#11806015
LR-Clonase II enzyme	Invitrogen	#11791020
Max Efficiency® DH10Bac™ competent E. coli	Invitrogen	#10361012
Cellfectin® II Reagent	Invitrogen	#10362-100
Grace's Insect Medium Unsupplemented	Gibco	#11595030
SFM4Insect™ with L-Glutamine	Cytiva,	# SH30912.01
Alexa Fluor 594 goat anti-mouse	Invitrogen	#A11032
VECTASHIELD mounting medium for fluorescence	Vector Laboratories	#H-1000
Terrific Broth	Gibco	#A13743-01
Ni-NTA His-Bind Resin	Novagen	#70666;
Mini-PROTEAN® TGX™	BioRad	# 456-1093
MINI-Protean TGX 14-15%, IPG/prep-well comb, 7cm IPG strip	BioRad	#456-1081
Chemiluminescence Blocking Reagent	Roche	#11500694001

BM chemiluminescence substrate solutions A and B	Roche	#11500694001
BugBuster Protein Extraction Reagent	Novagen	#70584-3
benzonase endonueclease	Novagen	#70664-3
Imject™ Alum	Thermo Scientific	#77161
Laemmli Sample Buffer	Bio-Rad	#1610747
12% SDS PAGE	Bio-Rad	#4561045
AddaVax™	InvivoGen	#vac-adx-10
assay buffer	Immunodiagnostics	#HS01
MagMAX mirVana Total RNA isolation Kit	applied biosystems by Thermo Fisher Scientific	#A27828
TRIzol™ Reagent	ThermoFisher Scientific	#15596026
RevertAid RT Kit	Thermo Fisher Scientific	#K1691
TaqMan 2x universal PCR master mix	applied biosystems by Thermo Fisher Scientific	#4304437
RBC Lysis Buffer	Biolegend	# 420301
Phytohemagglutinin (PHA)	Sigma Aldrich	#L8754
RPMI	Gibco	# 11530586
